# S161. FUNCTIONAL BRAIN NETWORKS INVOLVED IN ATTENTIONAL BIASING IN SCHIZOPHRENIA

**DOI:** 10.1093/schbul/sby018.948

**Published:** 2018-04-01

**Authors:** Paul Metzak, Todd Woodward

**Affiliations:** 1 University of Calgary; 2 University of British Columbia

## Abstract

**Background:**

Although the symptomatology in schizophrenia is variable, many of the cognitive deficits that are associated with the illness, including impairments in attention, working memory, verbal learning and executive functions, persist over time from the prodrome to the chronic phase. One of the cognitive domains showing pronounced deficits is executive function, which is the ability to adaptively adjust behavior in the face of changing environmental demands. Attentional biasing is one aspect of executive function that attentuates conflict between competing stimuli (or competing features of a stimulus) via the top-down regulation of attention. The goal of this study was to use functional magnetic resonance imaging (fMRI) to isolate the brain activity related to differences in levels of attentional biasing in schizophrenia patients, where these levels were varied from trial-to-trial by manipulating the number of relevant stimulus dimensions.

**Methods:**

Participants - Twenty-three schizophrenia patients and twenty-one healthy volunteers, matched on age and gender, were recruited from the Vancouver area.

Task – The task involved performing three discrete tasks in alternation: judging whether shapes are blue or red, judging whether numbers are odd or even, and judging whether letters are uppercase or lowercase. Each stimulus contained either one dimension that cued a task in the task set (e.g. the numeral ‘2’ in white ink), two dimensions (e.g. the numeral ‘2’ in blue ink), or three dimensions, such that all three tasks in the set are cued (e.g. the word ‘TWO’ written in blue ink). Each stimulus was presented in the center of the screen and the judgment to be performed was cued with a single word followed by a question mark.

**Results:**

The fMRI data was analyzed using Constrained Principal Component Analysis, which identifies brain networks common to all participants and indexes the activity of each network for each participant. Three components were extracted for further examination.

Component 1 displayed activations located in the visual cortices, parietal lobes, primary motor areas, supplementary motor area (SMA), dorsal anterior cingulate cortex (dAcc), and cerebellum. The statistical analysis indicated that this component was reliable but did not differentiate between patients and volunteers.

Component 2 displayed activations in the occipital lobes, dAcc, SMA, parietal lobes and primary motor areas, and deactivations in the medial prefrontal cortices and the posterior cingulate/precuneus. The statistical analysis indicated that the activity in this component was reliable, and became stronger as stimulus dimensions increased. However, the patients did not increase activity to the same degree as the volunteers in the most challenging condition.

Component 3 displayed activations in the occipital lobes, hippocampi, and left parietal and primary motor areas as well as deactivations in superior and middle frontal gyri. The statistical analysis indicated that this component was reliable, but activity levels did not differentiate between patients and volunteers.

**Discussion:**

The results indicate that patients and volunteers activated the same networks while performing the attentional biasing task. However, the statistical analysis of Component 2 suggests that patients display an inefficient pattern of brain activity, such that they have higher levels of activity than volunteers when little attentional biasing is required and significantly lower levels of activity than volunteers when high levels of attentional biasing was required. This pattern of results is suggestive of inefficient neural activity, particularly at higher levels of task difficulty, a finding which has previously been described in the schizophrenia literature.

